# Idiopathic autoimmune hemolytic anemia due to lecithin overdose: a case report

**DOI:** 10.1186/1757-1626-2-97

**Published:** 2009-01-29

**Authors:** Ioannis Lentzas, Panagiotis Papagiannopoulos, Ioannis Nikolaidis, Vasiliki Garmiri, Demosthenes Vatides, Athanasia Papathanasiou, Andreas Melidonis, Georgios Tsiros, Panagiota Voila, Christos Lionis

**Affiliations:** 1 Diabetes Center, Tzanion General Hospital, Piraeus, Tzani-Afentouli 1, 113-64 Greece; 2 Clinic of Social and Family Medicine, Department of Social Medicine, University of Crete, Heraklion, Crete, Greece; 3 Primary Health Care Post of Koukos, PHCC of Aiginio, Katerini, Greece; 4 Primary Heath Care Center of Gastouni, Ileia, Greece; 5 Microbiological Laboratory, Amaliada, Ileia, Greece; 6 Primary Health Care Post of Andravida, PHCC of Gastouni, Ileia, Greece

## Abstract

**Introduction:**

Idiopathic Autoimmune Hemolytic Anemia is a potentially fatal condition which requires prompt and potent treatment. Diagnosis of idiopathic autoimmune hemolytic anemia requires both serologic evidence of autoantibody presence and hemolysis. Although most of the times it is considered idiopathic, several underlying causes have been identified, like autoimmune and connective tissue diseases, viral infections, drugs or hyper function of the immune system. To our knowledge, this is the first case in the international literature describing lecithin-induced autoimmune hemolytic anemia.

**Case Presentation:**

This case report is to highlight a rare but dangerous adverse reaction to overdose of lecithin. A 38 year old white female from Greece, presented to our emergency room with progressive fatigue over a period of ten days and icteric discoloration of her skin and conjunctiva. The patient had been taking lecithin supplements (1200 mg, 3 capsules a day) over a period of ten days for weight loss. She reports that the last 3 days, prior to the examination, she took 5 capsules/day, so that the supplement would take effect more rapidly. Her past medical, social and family history showed no disturbance. Relatives of the patient were requested to submit any blood-tests taken over a period of 20 days prior to the onset of symptoms caused by Lecithin. All tests proved that all functions were within normal scale. Her physical examination revealed pallor and jaundice without palpable hepatosplenomegaly. Blood biochemistry tests showed total bilirubin 7.5 mg/dl, with indirect bilirubin 6.4 mg/dl and complete blood count showed hemoglobin 7.6 g/dl with blood levels 21.4%.

**Conclusion:**

In every case of idiopathic autoimmune hemolytic anemia the administration of pharmaceutical substances should always be examined, except for the standard reasons that cause it. In this case the cause of hemolysis was attributed to the excessive intake of lecithin capsules for the loss of body weight. It is important that clinicians and immunologists are aware of this adverse effect.

## Introduction

Idiopathic Autoimmune Hemolytic Anemia is a potentially fatal condition which requires prompt and potent treatment. Diagnosis of idiopathic autoimmune hemolytic anemia (IAIHA) requires both serologic evidence of autoantibody existence and hemolysis. Sensitized red blood cells (RBCs) are destroyed by intravascular and/or extravascular hemolysis. Although most of the times it is considered idiopathic (the cause of autoimmunization remains obscure in 50% of the patients) [1], several underlying causes have been identified, like autoimmune and connective tissue diseases, viral infections, drugs or malignancies of the immune system [2]. In the absence of a clinically apparent underlying disorder, testing for the presence of antinuclear antibodies, a serum protein electrophoresis (SPE) and a Computer Tomography (CT)-scan must be systematic. Conversely, if no abnormalities are revealed, the relevance of a systematic medullar biopsy (MB) at AIHA onset seems very low [3]. Phospholipids, which include the compound phosphatidyl choline, the chemical name for lecithin are lipids containing a phosphoric acid residue. The main phospholipids in lecithin from soya and sunflower are phosphatidyl choline, phosphatidyl inositol, phosphatidyl ethanolamine, and phosphatidic acid. Although lecithin was first observed and isolated from egg yolk in 1846, it was only 20 years later that the choline component in lecithin was identified [2]. The role of polyunsaturated fatty acids in decreasing the cholesterol levels in the blood is well founded in literature. In addition, dietary phospholipids from various origins, particularly polyunsaturated fatty acid phospholipids, such as those of soy bean lecithin have a cholesterol-lowering effect in humans [4]. There are studies which exhibit that soy-derived lecithin has significant effects on lowering cholesterol and triglyceride, while increasing high density lipoproteins (HDL) levels in the blood. [5,6]. This case study is to highlight a rare but hazardous adverse reaction to lecithin or to the other substances that the capsules contain. Consent was obtained from the patient for publication of the hereby study. The legal authorities have been notified of the adverse effect of over consumption of lecithin.

## Case presentation

**Figure 1:**
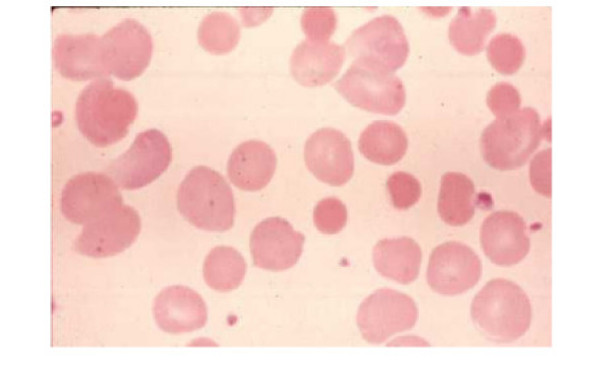
**The peripheral blood smear showing spherocytes**.

**Figure 2:**
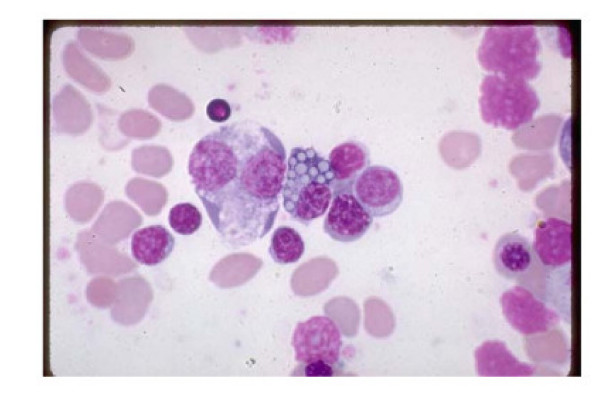
**Bone marrow biopsy**.

A 38 year old white female from Greece, presented to our emergency room with progressive fatigue over a period of ten days and icteric discoloration of her skin and conjunctiva. The patient had been taking lecithin supplements (1200 mg, 3 capsules a day) over a period of ten days for weight loss. Her medical, social and family history revealed no possible cause for such a condition. Relatives of the patient were requested to submit any blood-tests taken over a period of 20 days prior to the onset of symptoms caused by Lecithin. All tests proved that all functions were within normal scale: Haemoglobin 12.8 gr/dl, blood levels 39.3%, Mean cell volume (MCV) 84.2 fl, mean cell haemoglobin (MCH) 26.7 pg, mean cell haemoglobin concentration (MCHC) 32.5 gr/dl, White Blood Cell (WBC) 6400*1000/μl, Platelet counts (PLT) 210000*1000/μl, Urea 19 mg%, Creatinin 0.51 mg%, Lactate Dehydrogenase (LDH) 140 IU/L, Total bilirubin 0.78 mg%, Indirect bilirubin 0.19 mr%. Chemical and microscopic urine examination showed ph 5.5, s.w.1020, leukocytes 2–3 cells/h.p.f., erythrocytes 0–1 cells/h.p.f. and chemical and microscopic feces examination showed leukocytes 2–3 cells/h.p.f., erythrocytes 0–1 cells/h.p.f., without vermins. She denied the consumption of any medications, herbals or dietary supplements other than lecithin capsules during the past four weeks. She was not experiencing any shortness of breath or dyspnea on exertion. After her hospital intake physical examination revealed pallor and jaundice without palpable hepatosplenomegaly. No skin lesions or lymphadenopathy were observed following routine cardiac, pulmonary and abdominal examination. Blood biochemistry tests showed total bilirubin 7.5 mg/dl, with indirect bilirubin 6.4 mg/dl and increased LDH 405 IU/L. Liver function tests and the rest of the comprehensive metabolic panel (CMP) was within normal limits. Complete Blood Count showed haemoglobin 7.6 g/dl with blood levels 21.4%. MCV 99 fl, MCH 29.6 pg and MCHC 29.5 g/dl. White Blood Cell and Platelet counts were normal. Reticulocyte count was 15%. The peripheral blood smear showed spherocytes. (Fig. 1). Direct Antiglobulin Test (DAT) was positive for anti-IgG, but tests for anti-nuclear antibodies (ANA) and Rheumatoid Factor were negative, Thyroid function tests(T3 1.72 ng/ml, T4 9.45 μg/dl, TSH 2.07 μIU/ml) and Serum Protein Electrophoresis (Albumines 61%, a1 2.8%, a2 9.5%, β 10.4%, γ 13.2%) were unremarkable. C3 complement (0.99 gr/l), C4 (0,05 gr/l) and IgG (16,50 gr/l) were within normal rates. CT scan of the chest, abdomen and pelvis were unremarkable. Finally, bone marrow biopsy that was performed can be seen in (Fig 2). The patient was prescribed for prednisolone 60 mg per day. Her clinical picture during the 10-day hospitalization was uneventful. The patient was re-examined after four weeks, and her clinical and biochemical profile returned to normal without residual deficits or complications. She denied any consumption of lecithin, over this period; therefore our medical team did not conduct any further research.

## Discussion

Idiopathic autoimmune hemolytic anemia results from auto antibodies' production. Depending on the special characteristics of those antibodies, there are different types of autoimmune hemolytic anemia, a) Cold agglutinin disease, b) Warm agglutinin disease c) Donath-Landsteiner disease and d) drug-induced where auto antibodies are Ig-G type.

There are different underlying mechanisms of drug-induced autoimmune hemolytic anemia [7].

1. Drug-absorption. An epitope of the drug (lecithin) attach to the proteins of the erythrocyte membrane and finally leads to IgG antibodies formations.

2. Immune Complex Formation. When the substance combines with the plasma protein, it creates a complex which triggers the formation of antibodies against it. The new immune complex attaches to the RBC membrane and activates the complement which consequently leads to intravascular haemolysis.

3. The drug affects immune cells, so that it causes immune answer: DAT positive.

4. Probably the drug changes RBC membrane and that makes it vulnerable to the complement but nonspecifically, so that RBC's are destroyed extravascularly and that results in a negative DAT. [7].

In everyday clinical practice it is sometimes difficult to define the exact mechanism with which a pharmaceutical substance causes hemolysis, because more than one of the mechanisms that are mentioned before is involved. Fundamental step to the differential diagnosis of autoimmune hemolytic anemia is the exclusion of the remaining pathological conditions that cause it such as lymphohyperplastic disease, neoplasia, connective tissue disorders, thyroid gland disease, chronic hepatitis, and infections such as Ebstein-Barr Virus (EBV), Cytomegalovirus (CMV), Human Immunodeficiency Virus (HIV) and Mycoplasma.

In the present case all the above were excluded based on the history, the objective examination, and the laboratory findings. Consequently, the cause of autoimmune haemolytic anemia due to the administration of overdose of lecithin (or to the other components of the capsule) is very probable. The causing mechanism which is based on the positive DAT as antibody Ig-G and the action of lecithin as a drug absorption mechanism, or antibodies induction due to modification of RBC membrane by lecithin.

## Conclusion

To our knowledge this is the first case in the international literature describing lecithin-induced idiopathic autoimmune hemolytic anemia. Our treatment was in compliance with the recommended standard treatment of hemolytic anemia, with the discontinuation of the pharmaceutical agent, and namely the discontinuation of lecithin capsules, and the beginning of treatment with corticoids and it was efficient. It is important to the clinicians to be aware of this adverse effect and conduct more research. In every case of autoimmune hemolytic anaemia the administration of pharmaceutical substances should always be examined detached from the standard reasons that cause it. In this case the cause of hemolysis was attributed to the intake of an overdose of lecithin capsules for the loss of body weight.

## Competing interests

The authors declare that they have no competing interests.

## Authors' contributions

LI analyzed and interpreted the patient data regarding the hematological disease. PP collected the data and was a major contributor in writing the manuscript. NI performed the bone marrow biopsy. GV, VD, TG, VP and PA were contributors in writing the manuscript. LC interpreted the patient data regarding the hematological disease. MA performed and analyzed the auto-immune tests.

## Consent

Written informed consent was obtained from the patient for publication of this case report and accompanying images. A copy of the written consent is available for review by the Editor-in-Chief of this journal.
